# Epidemiology and mortality predictors of acute poisoning in a Tunisian Emergency Department: a two-year retrospective study

**DOI:** 10.1016/j.afjem.2026.101016

**Published:** 2026-09-18

**Authors:** Samia Bettout, Insaf Dlala, Nawel Farhat, Fatma Ben Salem, Achraf Belkacem, Sarra Kbaier, Meriem Mzahma, Amira Sghaier, Nahla Jerbi

**Affiliations:** a Faculty of Medicine of Monastir, University of Monastir, Monastir, Tunisia; b Emergency Department, Research Unit "Douleur thoracique", Tahar Sfar University Hospital, Mahdia, Tunisia

**Keywords:** Acute poisoning, Emergency department, Epidemiology, Mortality, Pesticides, Tunisia

## Abstract

**Introduction:**

Acute poisoning is a major public health challenge and a frequent cause of emergency department (ED) admission in Tunisia and North Africa. This study aimed to describe the epidemiological, clinical, and therapeutic characteristics of acute poisoning cases and to identify factors associated with mortality.

**Methods:**

A retrospective, single-center study was conducted at the ED of Taher Sfar University Hospital, Mahdia, from January 2022 to January 2024. All patients aged ≥12 years admitted for acute poisoning were included. Variables reflecting in-hospital interventions were excluded from the multivariate model to avoid reverse causality; only variables available at ED presentation were considered as candidate predictors. Multivariate logistic regression identified factors associated with in-hospital mortality.

**Results:**

Among 7645 ED admissions, 234 cases (3.06%) were for acute poisoning. The mean age was 30 years, with a female predominance (67.5%). Intentional poisoning accounted for 86.3% of cases. Pharmaceuticals (51.7%) and pesticides (30.3%) were the most common agents. The overall mortality rate was 12.8%, highest with aluminium phosphide (68.4%). Multivariate analysis identified three independent predictors of mortality, all available at ED presentation: altered mental status (GCS ≤ 8) (aOR=8.9, 95% CI 3.2–24.7), tachypnea (aOR=9.4, 95% CI 3.2–27.6), and poisoning by aluminium phosphide (aOR=10.2, 95% CI 3.5–29.7).

**Conclusion:**

Acute poisoning in this cohort predominantly involves intentional self-harm by young women, with high mortality linked to specific toxic agents and early clinical severity markers. These findings highlight the need for enhanced mental health services, stricter regulation of highly toxic pesticides, and optimized emergency care protocols in similar settings.

**African Relevance**

• Intentional self-poisoning among young women (86.3%) mirrors patterns across Africa.
• Pesticides caused 30.3% of cases and nearly half of deaths in agricultural communities.
• Simple clinical predictors offer practical triage tools for resource-limited African EDs.
• Low toxicology rates (15.8%) underscore the need for poison control systems in Africa.

## Introduction

Acute poisoning constitutes a major global public health challenge, leading to significant morbidity, mortality, and healthcare resource utilization worldwide [1]. The epidemiological profile varies considerably by geographic region, socio-economic context, and availability of toxic agents [2]. In low- and middle-income countries, the burden is disproportionately high, with mortality rates markedly exceeding those in high-income nations [3].

In north Africa, acute poisoning is a frequent and severe reason for Emergency Department (ED) admission. However, comprehensive national data remain scarce. In Tunisia, existing studies are often limited to specific populations or single centres, preventing a clear understanding of the national landscape [[4], [5], [6], [7]]. This knowledge gap hinders the development of effective prevention strategies and clinical management protocols tailored to local realities.

The ED of Taher Sfar University Hospital in Mahdia serves a large and diverse catchment area encompassing both urban and rural populations in central-eastern Tunisia, making it a representative site for characterizing acute poisoning patterns in a mixed demographic context. This study aimed to describe the epidemiological, clinical, and therapeutic characteristics of acute poisoning cases presenting to our ED and to identify factors associated with in-hospital mortality among these patients.

## Methods

### Study design and setting

A retrospective cohort study was conducted at the Emergency Department of Taher Sfar University Hospital, Mahdia, Tunisia, over 25 months (January 2022 – January 2024). The ED is staffed by emergency physicians 24/7, with on-site ICU consultation and a dedicated 12-bed medical ICU. Standard antidotes are stocked, but confirmatory toxicological testing is not available on-site.

### Study population

All consecutive patients aged ≥12 years presenting with a primary diagnosis of acute poisoning were eligible. The diagnosis was defined by clinical assessment, patient history, and confirmatory toxicology when available. Exclusions were: acute ethanol intoxication as the sole agent, envenomations, adverse drug reactions, patients leaving against medical advice, and incomplete medical records.

### Data collection

Data were retrospectively collected from medical records using a standardized form, capturing demographics, medical history, exposure characteristics (agent, route, circumstances), clinical findings on admission (vital signs, Glasgow Coma Scale), laboratory tests, management, and outcomes.

### Statistical analysis

Data were analyzed using IBM SPSS Statistics version 23.0 (IBM Corp., Armonk, NY, USA). Descriptive results were expressed as mean ± SD, median (IQR), or frequencies (%). Patients were dichotomized into survivors and non-survivors. Univariate comparisons used appropriate parametric or non-parametric tests, and Chi-square or Fisher's exact test for categorical variables, as appropriate.

To avoid reverse causality and time-dependent bias, variables reflecting in-hospital interventions or complications (mechanical ventilation, vasopressor support, acute kidney injury) were excluded from the multivariate model. Only variables available at ED presentation were considered as candidate predictors: age, sex, Glasgow Coma Scale (GCS) ≤ 8, tachypnea (respiratory rate > 20/min), hypotension (systolic blood pressure < 90 mmHg), toxic agent categories (pharmaceuticals, pesticides including aluminium phosphide and alpha-chloralose, caustic products, carbon monoxide), and psychiatric history.

Altered mental status was defined as a GCS score ≤ 8, consistent with the definition of severe coma requiring airway protection. Tachypnea was defined as a respiratory rate > 20/min, a threshold commonly used in emergency triage to identify patients at risk of respiratory compromise.

Variables with a p-value < 0.20 in univariate analysis were entered into a binary logistic regression model using a forward stepwise (likelihood ratio) selection method. The final model was restricted to three predictors to prevent overfitting, given the limited number of mortality events (n = 30), respecting the rule of thumb of at least 10 events per predictor variable. Multicollinearity was assessed using variance inflation factor (VIF), with a threshold of < 5 considered acceptable. Model calibration was evaluated using the Hosmer–Lemeshow goodness-of-fit test (p > 0.05 indicating adequate fit), and discrimination was assessed by the area under the receiver operating characteristic curve (AUC). Adjusted odds ratios (aOR) with 95% confidence intervals (CI) were reported. Statistical significance was set at p < 0.05.

### Ethical considerations

The study protocol received approval from the Institutional Ethics Committee of Taher Sfar University Hospital, Mahdia (Reference: CEM-20,251,206). Individual informed consent was waived due to the retrospective design.

## Results

**Fig. 1:**
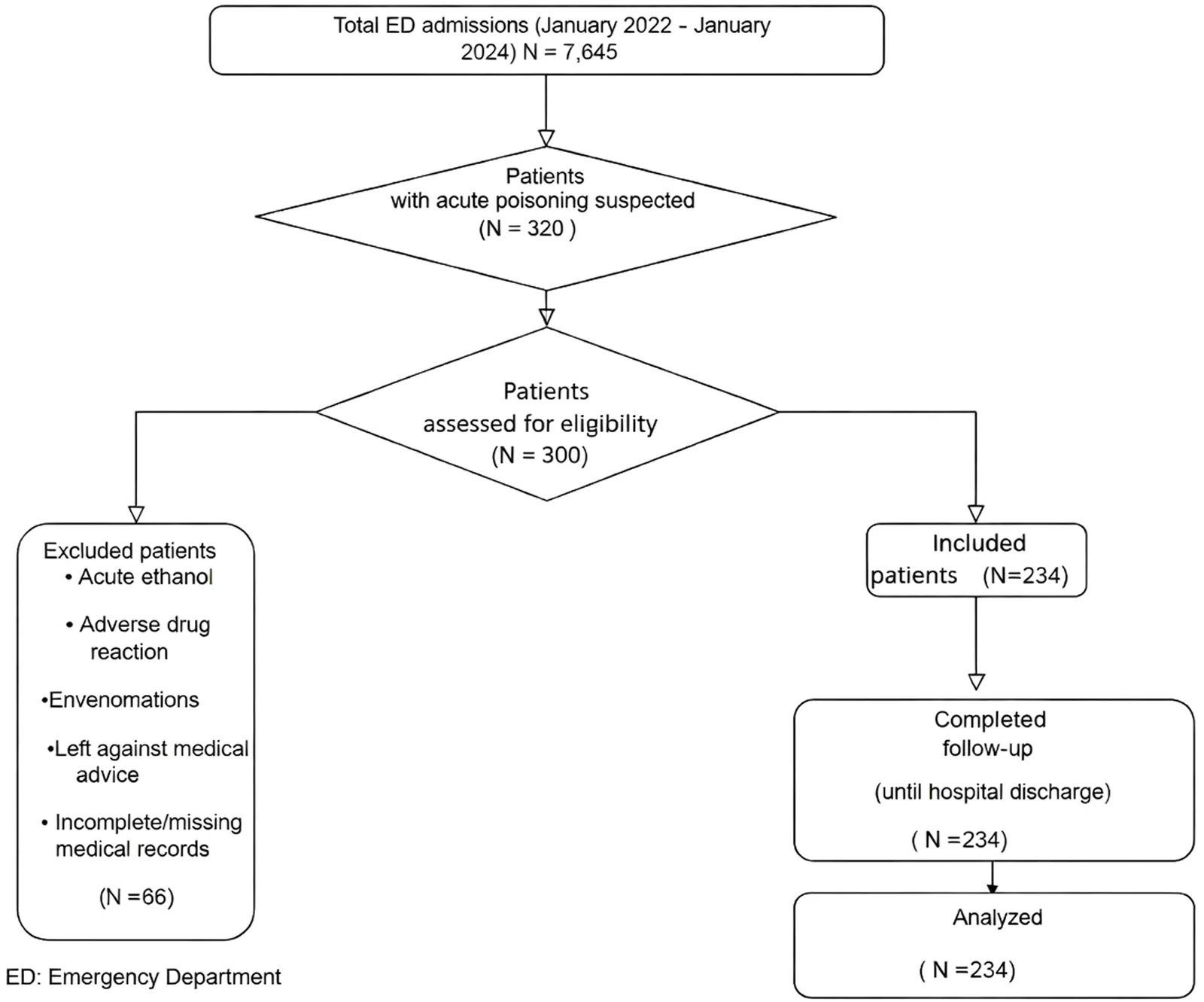
Patient flow diagram.

After exclusions, 234 patients were included, representing 3.06% of 7645 total ED admissions (Fig. 1).

The mean age was 29.97 ± 12.46 years, with the 20–40 year age group most affected. Female predominance was observed (158, 67.5%). Psychiatric history was present in 52 patients (22.2%), most commonly depressive disorders, and 50 (21.4%) had a previous suicide attempt (Table 1).

**Table 1:** Epidemiological characteristics and poisoning events (N = 234).

Variable	N (%)
**Gender**	
Female	158 (67.5)
Male	76 (32.5)
**Age group (years)**	
≤ 20	58 (24.8)
21–40	135 (57.7)
> 40	41 (17.5)
Psychiatric history	52 (22.2)
Previous suicide attempt	50 (21.4)
**Circumstance**	
Intentional	202 (86.3)
Unintentional	32 (13.7)
**Toxic agent**	
Pharmaceuticals	121 (51.7)
Pesticides	71 (30.3)
Caustic products	22 (9.4)
Carbon monoxide	15 (6.4)
Other / unknown	5 (2.1)

*N: total number of patients; ED: Emergency Department*.

Intentional poisoning (suicide attempt) was the leading circumstance (202, 86.3%). The oral route was involved in 93.1% of cases. Pharmaceuticals were the most common agents (121, 51.7%), followed by pesticides (71, 30.3%). Among pesticides, alpha-chloralose (12.8%) was predominant, followed by organophosphates (9.4%) and aluminium phosphide (8.1%). Other agents included caustic products (9.4%) and carbon monoxide (6.4%). No cases of combined poisoning (e.g., drugs and alcohol, or multiple toxic agents) were identified in our cohort. Median time from exposure to ED presentation was 2 h (IQR: 0.5–4) (Table 1).

Among the 121 pharmaceutical poisonings, psychotropics were the most frequently implicated class (67 cases, 55.4%), with benzodiazepines accounting for 42 cases (34.7%), antidepressants for 15 cases (12.4%), and neuroleptics for 10 cases (8.3%). Cardiovascular drugs (beta-blockers, calcium channel blockers, digoxin) were involved in 21 cases (17.4%), analgesics/NSAIDs (paracetamol, aspirin, ibuprofen) in 15 cases (12.4%), antiepileptics in 8 cases (6.6%), and other medications (antibiotics, antihistamines, antidiabetics) in 10 cases (8.2%).

Gastrointestinal symptoms (83.8%), neurological signs (78.2%), and cardiovascular signs (54.3%) were the most frequent manifestations. Common laboratory abnormalities included hyperleukocytosis (27.8%), metabolic acidosis (22.3%), and acute kidney injury (15.0%) (Table 2).

**Table 2:** Main clinical and biological findings (N = 234).

Variable	N (%)
**Clinical presentation**	
Gastrointestinal symptoms	196 (83.8)
Neurological signs	183 (78.2)
Cardiovascular signs	127 (54.3)
Tachypnea	89 (38.0)
Glasgow Coma Scale, mean ± SD	13 ± 3
**Laboratory abnormalities**	
Hyperleukocytosis	65 (27.8)
Metabolic acidosis	52 (22.3)
Acute kidney injury	35 (15.0)
Elevated troponin Ic	17 (7.3)

*SD: standard deviation*.

All patients received symptomatic treatment. Gastric lavage was performed in 37.2% and activated charcoal in 6.8%. Specific antidotes were administered to 7.7% of patients. Regarding caustic product poisoning, upper gastrointestinal endoscopy was performed within 12 to 24 h in 12 out of 22 patients (54.5%) based on clinical severity and symptoms (persistent odynophagia, dysphagia, or hematemesis). Mechanical ventilation was required for 23.5% and vasopressor support for 16.2%. Psychiatric evaluation was requested for 160 patients (68.4%).

Most patients were managed exclusively in the ED (69.4%), with ICU admission required for 9.9%. The mean length of stay was 1.89 ± 2.06 days. Complications occurred in 20.9% of patients, most commonly shock (7.7%). Thirty patients died, yielding an overall mortality rate of 12.8%. Mortality was highest with aluminium phosphide (68.4% case fatality), followed by alpha-chloralose (26.7%) and carbon monoxide (26.7%).

The median time from exposure to ED presentation was 2 h (IQR: 0.5–4) for survivors and 1.5 h (IQR: 0.5–3) for non-survivors, with no statistically significant association with mortality (p = 0.31).

Detailed information regarding previous suicide attempts (including the agent used and psychiatric follow-up status) was inconsistently documented in the medical records and could not be analyzed systematically.

Univariate analysis revealed that deceased patients more frequently presented with altered mental status (96.7%vs. 21.1%, p < 0.001), tachypnea (100%vs. 17.2%, p < 0.001), hypotension (53.3%vs. 5.9%, p < 0.001), and lower GCS (10 ± 3 vs. 13 ± 2, p < 0.001). Aluminium phosphide poisoning was strongly associated with mortality (43.3%vs. 2.9%, p < 0.001) (Table 3).

**Table 3:** Univariate analysis of factors associated with mortality.

Variable	Survivors (n = 204)	Non-survivors (n = 30)	p-value
**Clinical presentation**			
Altered mental status	43 (21.1)	29 (96.7)	<0.001
Tachypnea	35 (17.2)	30 (100)	<0.001
Hypotension	12 (5.9)	16 (53.3)	<0.001
GCS, mean ± SD	13 ± 2	10 ± 3	<0.001
Acute kidney injury	1 (0.5)	12 (40.0)	<0.001
**Toxic agent**			
Aluminium phosphide	6 (2.9)	13 (43.3)	<0.001
Alpha-chloralose	22 (10.8)	8 (26.7)	0.018
Carbon monoxide	11 (5.4)	4 (13.3)	0.030

*GCS: Glasgow Coma Scale; SD: standard deviation*.

Multivariate logistic regression, restricted to variables available at ED presentation, identified three independent predictors of in-hospital mortality: altered mental status (GCS ≤ 8) (aOR=8.9, 95% CI 3.2–24.7, p < 0.001), tachypnea (respiratory rate > 20/min) (aOR=9.4, 95% CI 3.2–27.6, p < 0.001), and aluminium phosphide poisoning (aOR=10.2, 95% CI 3.5–29.7, p < 0.001) (Table 4).

**Table 4:** Multivariate analysis of factors associated with in-hospital mortality.

Variable	aOR	95% CI	p-value
Altered mental status (GCS ≤ 8)	8.9	3.2–24.7	<0.001
Tachypnea (RR > 20/min)	9.4	3.2–27.6	<0.001
Aluminium phosphide poisoning	10.2	3.5–29.7	<0.001

*aOR: adjusted odds ratio; CI: confidence interval; GCS: Glasgow Coma Scale; RR: respiratory rate*.

The Hosmer–Lemeshow test indicated good model fit (χ² = 5.84, p = 0.66), and the AUC was 0.89 (95% CI 0.83–0.95), demonstrating excellent discrimination.

## Discussion

This study provides a current epidemiological profile of acute poisoning in a Tunisian ED, revealing a predominance of young females attempting suicide with pharmaceuticals, yet high mortality driven by specific pesticides. These findings have implications for emergency care across north Africa and similar resource-limited settings.

The overwhelming majority of poisonings were intentional, predominantly affecting young women. This pattern aligns with regional reports from north Africa and the Middle East [[8], [9], [10], [11]] and likely reflects the intersection of mental health disorders, socio-economic stressors, and easy access to toxic agents. Over one-fifth of patients had a documented psychiatric history, predominantly depression. However, psychiatric evaluation was requested for only 68.4% of cases, representing a missed opportunity for secondary prevention. Integrated mental health support in EDs has been shown to reduce repeat self-harm ([12,13]), and strengthening psychiatric liaison services should therefore be a priority in similar settings.

Although pharmaceuticals were the most frequently involved agents (51.7%), with psychotropics (55.4%) being the predominant class particularly benzodiazepines [14] the highest mortality burden was attributable to pesticides. Aluminium phosphide, in particular, demonstrated extreme lethality despite accounting for only 8.1% of cases. Its case fatality rate of 68.4% is consistent with reports from India, Iran, and Egypt ([15,16]), and reflects the rapid onset of cardiovascular collapse and the absence of an effective antidote [17]. The continued availability of such highly toxic pesticides in Tunisia represents a critical policy failure. International experience demonstrates that banning or restricting the most dangerous pesticides can rapidly reduce suicide rates ([18,19]), and stricter regulation is urgently needed.

The overall mortality rate of 12.8% in our cohort is higher than that reported in some European and North American series, where rates typically range from 2% to 5% ([20,21]). Several factors may explain this discrepancy. First, the case mix in our study includes a high proportion of pesticide poisonings (30.3%), which carry significantly higher lethality than pharmaceutical overdoses. Second, the mortality rate for alpha-chloralose poisoning in our cohort (26.7%) was notably higher than the 5–10% reported in some European studies [21]. This may reflect differences in ingested doses, delayed presentation, limited availability of intensive care resources, or the lack of systematic toxicological confirmation ([22,23]). Additionally, our single-center setting at a tertiary referral hospital may have selected for more severe cases, as less severe poisonings may have been managed at primary care facilities [[24], [25], [26], [27], [28], [29], [30], [31]]. These factors collectively contribute to the higher observed mortality and underscore the need for improved emergency care protocols and resource allocation in similar settings.

The independent predictors of mortality identified in this study altered mental status (GCS ≤ 8) and tachypnea are simple clinical markers that can be readily assessed at ED presentation without laboratory support. These findings provide pragmatic triage tools for resource-limited settings where toxicological assays are unavailable, enabling early identification of high-risk patients who may benefit from immediate ICU referral and aggressive supportive care according to current management guidelines [[32], [33], [34], [35]]. Aluminium phosphide poisoning emerged as the strongest predictor, reinforcing the need for heightened vigilance and early intensive management in these cases.

The low rate of confirmatory toxicology (15.8%) reflects a common challenge in low- and middle-income countries, where cost and limited laboratory capacity restrict testing [36]. This underscores the urgent need for improved diagnostic capacity and the establishment of a national toxicovigilance system to monitor epidemiological trends and guide evidence-based policy ([37,38]).

### Limitations

Several limitations should be acknowledged. The single-center retrospective design limits generalizability to other regions or settings. The low rate of confirmatory toxicological assays (15.8%) may have introduced exposure misclassification and recall bias. The absence of combined intoxication cases in our cohort may be related to underreporting in medical records or the lack of systematic toxicological screening, which could have missed multidrug or combined alcohol-toxin exposures. Detailed information regarding previous suicide attempts (including the agent used and psychiatric follow-up status) was inconsistently documented in medical records, precluding a meaningful analysis of these factors. With only 30 mortality events, the multivariate model was restricted to three predictors to avoid overfitting, and unmeasured confounders may remain. The inclusion of patients aged ≥12 years combines adolescents and adults, who may differ in intentionality, substance use patterns, and outcomes. Finally, a national, multicenter study is needed to validate these findings and assess the generalizability of the proposed predictors.

## Conclusion

Acute poisoning accounted for 3.06% of ED admissions, predominantly affecting young women through intentional self-harm (86.3%). Pharmaceuticals (51.7%) and pesticides (30.3%) were the main agents. Overall mortality was 12.8%, with aluminium phosphide being the most lethal (68.4%). Independent predictors of mortality at ED presentation were altered mental status (GCS ≤ 8), tachypnea, and aluminium phosphide poisoning.

Our findings highlight the need for stricter regulation of highly toxic pesticides; enhanced mental health services integrated into emergency care, and improved triage protocols for resource-limited settings. Establishing a national toxicovigilance system is a public health priority to monitor trends and guide evidence-based prevention strategies. Multicenter prospective studies are needed to validate these predictors and evaluate the impact of targeted interventions.

## Dissemination of results

Results from this study were shared with staff members at the Emergency Department of Taher Sfar University Hospital, Mahdia, through an informal presentation. The findings will be presented at national and regional emergency medicine conferences.

## Author contributions

**SB, ID, NF**: Conceptualization and study design, data acquisition and curation, formal analysis and statistical interpretation, drafting and review of manuscript; **FBS, AB**: Conceptualization and study design, drafting and review of manuscript; **SK, MM**: data acquisition and curation, drafting and review of manuscript; **AS, NJ**: drafting and review of manuscript. All authors approved the final version and remain accountable for the contents.

## Declaration of generative AI use

During the preparation of this manuscript, the authors used DeepSeek and DeepL Write for language editing and grammar improvement. After using this tool, the authors reviewed and revised the generated content as needed and take full responsibility for the final content of the publication.

## Declaration of competing interest

The authors declared no conflicts of interest.
